# Small bowel obstruction in patients associated with immune checkpoint inhibitors combined with chemotherapy: Two case reports and a review

**DOI:** 10.1097/MD.0000000000044910

**Published:** 2025-10-03

**Authors:** Xuefen Lu, Liping Qian, Chaochao Yu, Qihao Zhou

**Affiliations:** a Department of Pharmacy, Huzhou Nanxun People’s Hospital, Huzhou, China; b Department of Oncology, Huzhou Nanxun People’s Hospital, Huzhou, China; c Cancer Center, Department of Medical Oncology, Zhejiang Provincial People’s Hospital, Affiliated People’s Hospital, Hangzhou Medical College, Hangzhou, Zhejiang, China.

**Keywords:** cancer, case report, chemotherapy, immune checkpoint inhibitors, small bowel obstruction

## Abstract

**Rationale:**

The broader application of immune checkpoint inhibitors (ICIs) has led to increased recognition of rare but clinically significant adverse effects (AEs), including small bowel obstruction (SBO). This case aims to elucidate the clinical features and management challenges of ICI-associated SBO.

**Patient concerns:**

The first case involves a 67-year-old male patient with esophageal malignancy who was hospitalized due to painful abdominal distension following the 2 cycles administration of tislelizumab in conjunction with nab-paclitaxel. The second case concerns a 59-year-old male patient with pancreatic malignancy who developed abdominal pain and distension after undergoing combination capecitabine with sintilimab.

**Diagnoses:**

Imaging confirmed incomplete SBO in the first patient, while the second showed SBO with abdominal effusion.

**Interventions:**

In consideration of the AEs associated with ICI therapy, the patient was treated with glucocorticoids. Initial management included fasting, and administration of cefoperazone–sulbactam for anti-infection therapy along with methylprednisolone. Another patient underwent catheter placement and received octreotide treatment, but the therapeutic response was unsatisfactory.

**Outcomes:**

The patient 1 symptoms gradually improved. However, despite therapy, the patient 2’s SBO did not improve, and he was subsequently transferred to the hospital for further management.

**Lessons:**

With expanding use of ICIs, immune-related AEs have emerged. Clinicians should remain highly vigilant for gastrointestinal symptoms in patients receiving ICIs, as early recognition of SBO is crucial for timely diagnosis and treatment.

## 1. Introduction

With the widespread use of immune checkpoint inhibitors (ICIs), an increasing number of adverse events (AEs) have emerged, with intestinal toxicity being of particular concern.^[1]^ Immune-related adverse events (irAEs) associated with PD-1 inhibitors most commonly manifest as colitis, with an incidence of approximately 1%, and typical gastrointestinal toxicity also included nausea, decreased appetite.^[2]^ However, it is noteworthy that small intestinal obstruction (SBO), although rare, has emerged as a complication of increasing concern. Cases of intestinal obstruction during treatment with nivolumab have also been reported by Tso et al^[3]^ and Fragulidis et al,^[4]^ suggesting its association with immune-mediated intestinal damage.

SBO is a common type of intestinal obstruction. It also is one of the common acute abdominal diseases in surgery, and the common clinical symptoms are abdominal pain, abdominal distension, nausea and vomiting, and the anus stops defecation.^[5,6]^ If not diagnosed and treated in time, it will cause water and electrolyte disorders, posing a serious threat to the patient’s life safety.^[7]^

The primary mechanisms of intestinal obstruction include surgical trauma, which triggers a stress response that reduces intestinal motility.^[8]^ Additionally, surgical anesthesia and opioids can stimulate opioid receptors in the gut, also leading to reduced intestinal motility and obstruction.^[9]^ This case report introduces ICI-mediated inflammatory gastrointestinal dysmotility. Unlike previous cases, immune-mediated pseudo-obstruction does not involve a structural lesion. It is similar to chronic intestinal pseudo-obstruction, a rare disorder characterized by gastrointestinal motility dysfunction.^[10]^

The mechanism of ICIs-associated intestinal toxicity is currently unknown, but some studies suggest that it may be related to the cytotoxicity of ICIs, leading to intestinal toxicity.^[11]^ ICIs can cause T-cell overactivation and increased production of inflammatory cytokines. This, in turn, activates the intestinal intrinsic immune system, resulting in the release of inflammatory factors that diminish intestinal motility.^[12]^ While AEs of SBO have been observed in certain clinical trials involving domestic ICIs, there have been a notable absence of pertinent reports in real-world settings. Here, we present 2 unusual cases in which SBO ensued, considered a result of an AE associated with ICIs in combination with chemotherapy. To our knowledge, this is the first real-world report on intestinal obstruction related to domestically produced ICIs. Notably, although nivolumab-related intestinal obstruction has been reported previously, this constitutes the first real-world evidence documenting this AE specifically for domestically produced ICIs, with detailed correlation and treatment response analysis.

## 2. Case presentation

### 2.1. Case 1

A 67-year-old male was diagnosed with unresectable esophageal squamous cell carcinoma (ESCC) in May 2020. He underwent 9 cycles of nab-paclitaxel and carboplatin chemotherapy combined with radiation therapy. However, 7 months later, he developed liver metastasis. Subsequently, he received 2 cycles of nab-paclitaxel, cisplatin, and camrelizumab treatment. Due to grade 3 anemia (hemoglobin 77 g/L) based on the WHO grading system, cisplatin was discontinued. The patient then continued with nab-paclitaxel and camrelizumab for an additional 5 cycles. After treatment, the patient had a stable disease.

Unfortunately, 3 months later, his CT and magnetic resonance imaging scans revealed tumors in the esophagus and liver enlargement. He underwent 6 cycles of treatment with a combination of docetaxel and camrelizumab, but antitumor treatment was suspended for 3 months due to the COVID-19 pandemic. After the tumor progressed again, he was given capecitabine combined with camrelizumab. However, due to skin itching, capecitabine was replaced with tegafur, gimeracil, and oteracil potassium capsules (S-1). Whether it was capecitabine or S-1, the patient often forgot to take the medication orally when he was at home. Considering disease progression and the importance of taking medication on time, the patient received treatment with nab-paclitaxel (200 mg on day 1) combined with tislelizumab (200 mg on day 1) for 2 cycles from November to December 2023.

**Figure 1.**
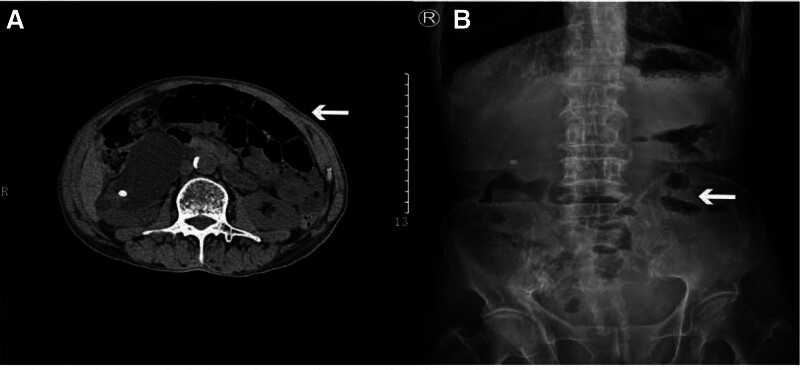
The imageological examination of case 1 patient. (A) Abdominal CT (taken on December 3, 2023) and (B) abdominal x-rays (taken on December 11, 2023) suggesting “intestinal obstruction.” CT = computed tomography.

Three days after the last treatment, the patient experienced chest tightness, shortness of breath, fatigue, a mild cough, abdominal distension, and pain. Laboratory examination revealed a hemoglobin level of 72 g/L (normal range: 130–175 g/L), hypersensitive C-reactive protein (hs-CRP) of 163.4 mg/L (normal range: 0.0–10.0 mg/L), and no significant abnormalities in white blood cells, platelets, creatinine, alanine aminotransferase, or aspartate aminotransferase. ANC 5.3 × 10^9^/L (normal range) at SBO onset. On physical examination, the vital signs were as follows: Body temperature, 36.5°C; blood pressure, 117/68 mm Hg; heart rate, 82 beats/min; respiratory rate, 19 breaths/min. Furthermore, clinical examination showed mild abdominal pain upon palpation. An abdominal CT scan indicated “incomplete intestinal obstruction of the small intestine” (Fig. 1A, B).

A 67-year-old male patient with ESCC developed abdominal pain and distension following treatment with tislelizumab combined with chemotherapy, with abdominal CT suggesting SBO (CTCAE v5.0 grade 3 due to radiographic evidence). Acute onset within 14 days post-ICI therapy, consistent with the typical window for irAEs. Based on a Naranjo score of 6, we highly suspect that this case of bowel obstruction is an adverse reaction to the combination of the tislelizumab and chemotherapy. CT revealed gas and fluid accumulation with bowel dilatation and air-fluid levels in the bowel segments proximal to the ileum in the right lower abdomen, accompanied by blurred mesenteric fat planes. Notably, no densely packed mass was identified in the bowel wall. Significantly elevated (hs-CRP: 163.4 mg/L, normal < 10.0 mg/L) accompanied by anemia (Hb 72 g/L), indicating systemic inflammation. Tomography/computed tomography scans before and after are still needed to rule out tumor recurrence.

Following discontinuation of anticancer therapy, the patient’s intestinal obstruction showed no progression. After 24 hours of steroid treatment, the patient’s VAS score decreased from 7 to 5. At 72 hours posttreatment, the VAS score further decreased to 3. Inflammatory markers, such as hs-CRP levels, also showed a reduction. This is consistent with the ASCO guidelines (2023).^[13]^ Combined with previous abdominal CT scans showing the obstruction was new onset, we can rule out the possibility of the bowel obstruction being caused by tumor metastasis. Combined with the patient’s medical history, the final diagnosis was SBO.

**Figure 2.**
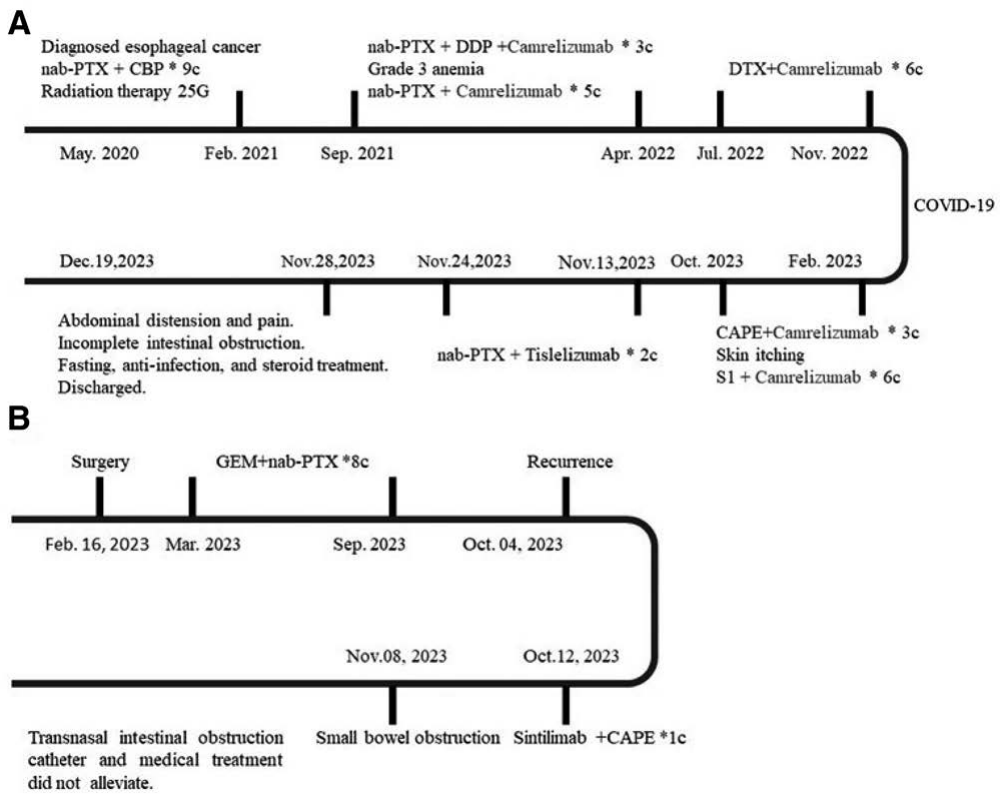
The treatment chart of the patients. (A) The treatment timeline of case 1 patient. (B) The treatment timeline of case 2 patient. CAPE = capecitabine, CBP = carboplatin, DDP = cisplatin, DTX = docetaxel, nab-PTX = nab-paclitaxel, GEM = gemcitabine, S1 = oteracil porassium capsules.

Initial treatment involves adjusting the dose of octreotide based on the daily nasogastric tube drainage, with a starting dose of 50 to 100 μg, 3 times daily. Immunosuppressive therapy begins with methylprednisolone at 1 mg/kg, followed by a gradual weekly dose reduction of 20%. Afterward, nutritional support is provided to establish intestinal tolerance. The patient’s condition improved, and he was discharged after 20 days of hospitalization. Figure 2A illustrates the course of his treatment. From the patient’s perspective, the symptoms of SBO were extremely uncomfortable. Prompt and meticulous treatment, coupled with attentive and compassionate care from the dedicated medical staff, effectively fostered a rapport with him, significantly contributing to the alleviation of his anxiety and promoting a sense of well-being.

### 2.2. Case 2

**Figure 3.**
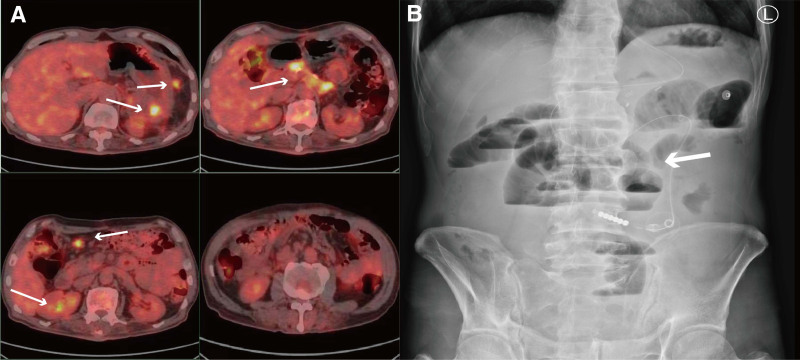
The imageological examination of case 2 patient. (A) The PET/CT (October 4, 2023) showed soft tissue thickening at the surgical site of pancreatic cancer, suggesting invasion of the adjacent gastric wall with increased FDG metabolism. (B) The CT scan (taken on November 8, 2023) of the abdomen further showed small bowel obstruction and fluid in the abdominal cavity. CT = computed tomography.

A 59-year-old man, with a history of smoking and alcohol consumption, was diagnosed with pancreatic cancer due to abdominal pain. On February 16, 2023, he underwent surgery that included a pancreatic subtotal resection, en-bloc splenectomy, intestinal adhesiolysis, perirenal adhesiolysis, and portal vein suturing. Postoperative pathology revealed an intermediate- to low-differentiated ductal adenocarcinoma of the pancreas, measuring 7.5 cm × 3.5 cm  × 2.8 cm, which had infiltrated the surrounding fibers, adipose tissue, and nerve. Immunohistochemical analysis showed hMLH1(+), hMSH2(+), hMSH6(+), PMS2(+), PD-L1(E1L3N)(CPS:<1). Based on these findings, he was classified as T4N1M0, corresponding to stage III of the disease. He was treated with a combination of gemcitabine and nab-paclitaxel for a total of 8 cycles, spanning from March to September 2023. Nevertheless, 1 month subsequent to his final chemotherapy session, he developed severe abdominal pain. The tomography/computed tomography scan revealed thickening of the soft tissue in the surgical site of the pancreatic cancer, indicating invasion of the adjacent gastric wall, accompanied by increased fluorodeoxyglucose (FDG) metabolism. This finding was suggestive of recurrence. Additionally, multiple nodules with elevated FDG metabolism were observed in the omentum, mesentery, and peritoneum, indicating metastasis and further invasion of the adjacent gastric wall. Moreover, enlarged retroperitoneal lymph nodes with increased FDG metabolism were also noted, which were considered indicative of metastatic disease (Fig. 3A).

He was admitted to our hospital in December 2023 due to abdominal pain, lower back pain, fatigue, and poor appetite. Following treatment, his symptoms showed improvement. However, despite our recommendation for a biopsy and genetic testing, he declined. Given his poor performance status (PS), we initiated a regimen of oral capecitabine (1.5 g administered twice daily for 14 consecutive days) in combination with intravenous sintilimab (200 mg on day 1).

Fifteen days after treatment, the patient presented to the hospital with complaints of abdominal pain, distension, and pain in the lower back. Laboratory examination revealed a hemoglobin level of 107 g/L, while hs-CRP, white blood cell count, platelet count, creatinine, alanine aminotransferase, and aspartate aminotransferase levels were all within normal limits. ANC 6.1 × 10^9^/L (mild neutropenia) during obstruction. On physical examination, the vital signs were as follows: Body temperature, 36.7°C; blood pressure, 115/82 mm Hg; heart rate, 92 beats/min; respiratory rate, 20 breaths/min. Clinical examination demonstrated moderate abdominal distension and pain upon palpation, but no peritoneal irritation or low back pain was evident. Abdominal ultrasound findings included a postoperative gallbladder and mild dilatation of the upper portion of the common bile duct. An abdominal CT scan further revealed SBO and the presence of fluid in the abdominal cavity (Fig. 3B).

A 59-year-old male pancreatic cancer patient developed abdominal pain and distension after receiving sintilimab (anti-PD-1 antibody) combined with chemotherapy. CT demonstrated SBO with significant fluid accumulation, predominantly involving the terminal ileum within 50 cm of the ileocecal valve. The imaging demonstrated 6-mm concentric wall thickening, while notably lacking evidence of mechanical obstruction points, volvulus, or peritoneal carcinomatosis. The patient with a history of postoperative chemotherapy, with no obvious signs of intestinal obstruction, developed significant abdominal pain and intussusception after the first cycle of ICI therapy, suggesting possible ICI-related intestinal toxicity.

Following completion of fasting and fluid replacement, the patient demonstrated poor response to conventional treatment regimens including octreotide therapy. Despite methylprednisolone administration exceeding 72 hours, the VAS score decreased only from 8 to 6. Serial laboratory tests indicated persistent inflammatory status. This AE was rated as grade 3 according to CTCAE v5.0 criteria. The Naranjo score was 6, and considering the association with medication administered 15 days prior and the relief experienced after steroid treatment, the event may be related to an ICI. Other supportive care measures included symptomatic treatment with paroxetine and pain management with a hydromorphone PCA at a basal rate of 0.2 mg/h. A dedicated pain management team is adjusting the dosage.

Following surgical consultation, it was deemed that laparotomy was not indicated due to the presence of tumor metastasis in the abdominal cavity and the patient’s poor PS. Consequently, he was transferred to another hospital 28 days after admission. Figure 2B illustrates the course of his treatment. From the patient’s perspective, he was experiencing great pain and discomfort, and he desperately hoped that a surgeon could find a way to help him.

## 3. Discussion

As a novel treatment strategy for cancer patients, ICIs, in contrast to traditional cytotoxic medications, have consequently brought significant clinical benefits and extended their overall survival. They achieve this by increasing the immune system’s capacity to destroy cancer cells, specifically by obstructing negative regulators that are expressed in tumors or immune cells.^[14]^ As of right now, Chinese domestically produced ICIs (camrelizumab, tislelizumab, sintilimab, and toripalimab), either alone or in combination with chemotherapy, are the accepted first- or second-line treatment for various cancers in China.^[15]^ What is more, domestic ICIs are priced significantly lower than their imported counterparts, including nivolumab, pembrolizumab, atezolizumab, and durvalumab.

Clinical trial studies have demonstrated that the combination of tislelizumab and chemotherapy for advanced or metastatic ESCC yielded superior overall survival and progression-free survival with a manageable safety profile, compared to placebo plus chemotherapy.^[16,17]^ Therefore, for the patient with ESCC in case 1, we chose to administer tislelizumab combined with chemotherapy for anti-tumor treatment. Although the effectiveness of ICIs is limited in pancreatic cancer, some studies suggest that combining sintilimab with chemotherapy may be a viable treatment option.^[18,19]^ Consequently, for the pancreatic cancer patient in case 2, we opted for sintilimab combined with chemotherapy as the anti-tumor treatment.

Some of the more common contributors to this phenomenon are chemotherapy and the combination of ICIs used in cancer treatment. Research has shown that the incidence of any grade AEs in the ICIs combination therapy group is significantly higher than in the ICIs monotherapy group.^[14]^ A retrospective examination in a single-arm cohort study revealed that patients with locally advanced non-small cell lung cancer experienced gastrointestinal events following the administration of a combination of tislelizumab and platinum-based doublet therapy. These included 1 case (2.56%) of nausea, 5 cases (12.82%) of vomiting, 6 cases (15.38%) of constipation (grade 1), and 4 cases (10.26%) of constipation (grade 2).^[20]^ Similarly, in the phase III KEYNOTE-189 trial, the incidence of gastrointestinal toxicity was significantly higher in the pembrolizumab–chemotherapy group compared to those receiving chemotherapy alone, suggesting pembrolizumab as a potential factor in this toxicity when combined with chemotherapy.^[21]^ The severity of enteritis induced by the combination of chemotherapy and ICIs is uncommon enough to result in rare cases of paralytic intestinal obstruction.^[22]^ Both of our cases have a history of using ICIs in combination with chemotherapy before the occurrence of intestinal obstruction. Chemotherapy drugs may cause paralytic intestinal obstruction due to their neurotoxicity, which can lead to intestinal dysfunction, oxidative stress, and the loss of intestinal neurons. However, based on the studies mentioned earlier, it is evident that the combined use of ICIs is associated with a higher incidence of intestinal toxicity. This heightened toxicity may stem from ICIs triggering excessive activation of T cells and subsequently boosting the production of inflammatory cytokines. This, in turn, activates the intestine’s intrinsic immune system, causing the release of inflammatory factors and a subsequent decrease in intestinal motility.^[12]^ Moreover, patients undergoing laparotomy almost always develop adhesions or adhesion bands.^[23]^ Although case 2 underwent surgery, there was no intestinal obstruction after the surgery; instead, intestinal obstruction occurred after recurrence and subsequent ICI combination treatment.

Malignant intestinal obstruction (MBO) is common in advanced cancer patients, and in clinical practice, we need to distinguish whether it is caused by ICIs or by the malignancy itself. MBO can be mechanical or functional,^[24]^ usually caused by tumor infiltration in the mesentery, nerves, and/or abdomen and intestinal plexus.^[25]^ It is difficult to distinguish them clinically, often relying on medication history and the order of occurrence. It is noteworthy that unlike mechanical intussusception, ICI-related cases may lack structural lesions but exhibit systemic inflammation (elevated hs-CRP) and rapid steroid response. VAS and biomarker trends provide dynamic evidence for treatment efficacy. The patient in case 2 developed multiple abdominal metastases and abdominal pain 1 month after completing postoperative chemotherapy, with the pain subsiding after our treatment. At that time, he did not exhibit any signs of intestinal obstruction. However, following a course of ICI treatment, he experienced severe abdominal pain and was subsequently diagnosed with SBO. Despite conservative management (including intestinal obstruction tube placement and supportive care), symptoms (abdominal pain and obstruction) showed no improvement. We suspect that his intestinal obstruction is closely related to the ICI therapy. Potential factors for treatment failure include the overlap of tumor-related inflammation and immune toxicity; furthermore, the overexpression of PD-L1 in intestinal macrophages may impair steroid efficacy by maintaining local immune activation; and the delay in initiating steroids could also impact effectiveness. The ANC levels, particularly in case 2 demonstrating mild neutropenia, provide additional insight into the immune status during irAE development.

The treatment options for MBO include extensive surgical and nonsurgical interventions. Surgery may be a necessary choice for patients with a clearly defined obstruction point and signs of possible ischemia.^[7]^ Single-stage surgery can relieve the obstruction and eradicate the cancer.^[26]^ Large retrospective reviews have shown that patients treated with surgery exhibit the longest survival rates, yet this should not automatically be interpreted as evidence that surgery is superior to medical or procedural management. Instead, this finding may reflect the tendency to perform surgery on patients who are in better health and have less severe conditions.^[7]^ For patients who are not surgical candidates, drug therapies such as painkillers, antiemetics, steroids, and antisecretory agents can be used.^[27]^ Octreotide is recommended for MBO patients who are not suitable for surgery.^[24]^ The Multinational Association of Supportive Care in Cancer guidelines recommend that when managing symptoms related to MBO in advanced cancer patients, supportive treatment measures such as nasogastric tube decompression and nutrition should be considered.^[25]^

We conducted a literature review of domestically produced ICIs (shown in Table 1) and found that although there are records showing that, although there are records indicating that domestic ICIs can result in AEs, such as intestinal obstruction, the incidence rate of these AEs is very low in clinical trials. In the phase I/II Study of single-agent tislelizumab in solid tumors, Desai et al reported 3 cases of SBO (grade 3) in patients receiving tislelizumab as a single agent.^[2]^ In other trial studies, Lin Shen et al^[28]^ and Jian Li et al^[29]^ reported, respectively, that 4 (1.3%) and 3 (3.8%) cases of grade ≥ 3 intestinal obstruction occurred after tislelizumab injection. Jiang et al identified 1 case (0.7%) of bowel obstruction following sintilimab treatment in a phase Ib study.^[32]^ In addition, in trial studies, the combined use of ICIs with other medications has also been noted to cause intestinal obstruction. In the phase 1a/Ib Trial of pamiparib combined with tislelizumab for advanced solid tumors, Michael Friedlander et al reported 2 (4%) cases of grade 3 SBO.^[30]^ The phase 2 study by Yang et al reported that patients first received tislelizumab and then underwent total mesorectal excision, which led to intestinal obstruction. It is worth noting that these were cases of intestinal obstruction involving the total mesorectal excision that occurred subsequent to ICI treatment. Their causes may still be related to the surgery.^[31]^ In a sintilimab phase 3 trial study, 1 case (1%) of intestinal obstruction, classified as grade 5, has been reported among the patients receiving combination chemotherapy with sintilimab and IBI305.^[33]^ One case of bowel obstruction was also reported after sintilimab + mFFX treatment, although no efficacy evaluation was provided.^[18]^ In all clinical trials of camrelizumab, only 1 case (4.17%) of intestinal obstruction was reported in a phase 2, single-arm prospective study.^[34]^

**Table 1:** Characteristics of intestinal obstruction in trail studies of Chinese domestically produced ICIs.

Publication	Type of study	ICIs	PT	Treatment regimen	Number of patients	Number of intestinal obstruction and grade
Desai et al^[2]^	Phase IA/IB study (NCT02407990)	Tislelizumab	Advanced solid tumors	Single-agent Tislelizumab	451	3 (0.7%): grade 3
Lin Shen et al^[28]^	Multicenter phase 1/2 study (CTR20160872)	Tislelizumab	Advanced solid tumors	Tislelizumab 200 mg Q3W	300	4 (1.3%)
Jian Li et al^[29]^	Ongoing, single-arm, open-label, multicenter study (RATIONALE-209; NCT03736889)	Tislelizumab	Previously treated, locally advanced unresectable or metastatic MSI-H/dMMR solid tumors	Tislelizumab 200 mg Q3W	80	3 (3.8%)grade ≥ 3
Michael Friedlander et al^[30]^	Dose-escalation stage of a multicentre, open-label, phase 1a/b trial (NCT02660034)	Tislelizumab	Advanced solid tumors	Cohorts 1–3: tislelizumab 2 mg/kg + pamiparib (20, 40, or 60 mg oral bid) Q3W; cohorts 4 and 5: tislelizumab 200 mg + pamiparib (40, or 60 mg oral bid) Q3W.	49	2 (4%): grade 3
Zhengyang Yang et al^[31]^	Multicenter phase 2 study (NCT04911517)	Tislelizumab	LARC, cT_3–4a_N_0_M_0_ and cT_1–4a_N_1–2_M_0_	The entire course of neoadjuvant (CRT plus 3 cycles + Tislelizumab) and further radical surgery	50	1 (2.2 %): grade 1–2, 1 (2.2 %), ≥grade 3
Haiping Jiang et al^[32]^	Phase Ib study	Sintilimab	Advanced solid tumors	Sintilimab monotherapy settings	146	1 (0.7%): grade 3–5
Shun Lu et al^[33]^	Randomised, double-blind, multicentre, phase 3 trial (ORIENT-31; NCT03802240)	Sintilimab	EGFR-mutated NSQ-NSCLC who progressed on EGFR-TKI	Sintilimab + IBI305 + chemotherapy	148	1 (1%): grade 5
Qihan Fu et al^[18]^	Randomized phase II CISPD3 Trial (NCT03977272)	Sintilimab	Metastatic or recurrent pancreatic cancer	Sintilimab + mFFX	55	1 (1.8%)
Chao Jing et al^[34]^	Phase 2, single-arm, prospective study (NCT04345783)	Camrelizumab	Advanced gastric or gastroesophageal junction adenocarcinoma	Camrelizumab 200 mg on day 1 + apatinib 500 mg oral Qd + S-1 in specific dose on day 1–14	24	1 (4.17%)

Bid = twice daily, CRT = chemoradiotherapy, EGFR = epidermal growth factor receptor, ICIs = immune checkpoint inhibitors, LARC = locally advanced rectal cancer, mFFX = modified FOLFIRINOX, MSI-H/dMMR = microsatellite instability-high/mismatch repair-deficient, NSQ-NSCLC = non-squamous non-small-cell lung cancer, PT = pathological types, Q3W = every 3 weeks, Qd = once daily, TKI = tyrosine-kinase inhibitor.

To our best knowledge, we are reporting for the first time intestinal obstruction induced by domestic ICIs in combination with chemotherapy in the real world, suggesting a potential association between ICI-based chemotherapy and gastrointestinal complications. However, our report is not without limitations. Firstly, as neither case underwent an exploratory laparotomy, it was impossible to obtain pathological evidence for confirmation. Secondly, the intestinal obstruction catheter and conservative medical treatment administered to the patient in case 2 did not alleviate his abdominal pain or resolve his intestinal obstruction, indicating that he would need to undergo surgical treatment. However, after thorough consultation with our hospital’s surgical department, taking into account his tumor metastases and poor PS, the decision was made to refrain from surgery. This is indeed a great pity.Furthermore, due to the relative rarity of immune-related intestinal obstruction in clinical practice, even when patients present with clear precipitating factors such as a history of abdominal surgery, adequate prevention and identification often remain inadequate in the initial stages. Only after systematic investigation is the immune etiology confirmed and targeted treatment initiated. Although initial treatment yields some efficacy, the lack of long-term follow-up for patients prevents the acquisition of critical follow-up examination and prognostic data, thereby limiting our comprehensive assessment of the disease.

## 4. Conclusion

In summary, we report a rare but serious complication of SBO induced by the combination of domestically produced ICIs and chemotherapy in cancer treatment. This complication has a very low incidence observed in clinical trials, and we present the first relevant report in the real word. During the clinical use of ICIs, clinicians and pharmacists should vigilantly monitor for adverse reactions, enabling early identification, prompt diagnosis, and effective management of such rare AEs. For suspected SBO patients, enhanced CT should be prioritized. This finding fills the gap in real-world toxicity data for domestically produced ICIs, indicating that future management strategies need optimization via regional irAE registries and selective gut immune modulators. These cases highlight that ICI-related SBO requires prompt decompression, early steroids, and sustained bowel rest until CRP normalizes.

## Acknowledgments

We thank the patients for providing the necessary medical history required for this case report. We thank Jingru Xie for her valuable contributions to this study prior to her institutional transition. At her request, she has been removed from the authorship list due to affiliation changes.

## Author contributions

**Conceptualization:** Liping Qian.

**Data curation:** Chaochao Yu.

**Investigation:** Xuefen Lu, Chaochao Yu.

**Methodology:** Xuefen Lu.

**Project administration:** Qihao Zhou.

**Resources:** Qihao Zhou.

**Supervision:** Liping Qian.

**Visualization:** Xuefen Lu.

**Writing – original draft:** Xuefen Lu, Qihao Zhou.

**Writing – review & editing:** Liping Qian, Chaochao Yu, Qihao Zhou.
